# Veterinary Medical Society of the Ontario Veterinary College

**Published:** 1898-03

**Authors:** C. W. Fisher

**Affiliations:** Secretary


Secretary.

### VETERINARY MEDICAL SOCIETY OF THE ONTARIO VETERINARY COLLEGE.

THE regular weekly meetings of the Society were very interesting during the past month. The papers were well written, and many valuable points were brought forward during the discussions. As every student is required to be with a practitioner during the summer vacation, the methods of many eminent veterinarians are in this way communicated to the students. They therefore find the meetings of great value in obtaining both theoretical and practical knowledge. The following is a list of the papers read: P. LeC. Garnett, "Chloroform as an Anæsthetic;" J. S. McIntyre, "Craniotomy in Cow;" L. Bailey, "Nasal Gleet in Sheep;" A. G. Van Tine, "Abortion;" E. H. Lawley, "Anthrax;" J. G. Cruikshanks, "Indigestion in Horse;" H. P. Reed, "Sanitas;" B. W. Powell, "Modes of Death;" C. H. A. Stevenson, "Open Joint;" W. A. Campbell, "Periodic Ophthalmia;" J. Dixon, "Aconite;" W. R. Clark, "Means of Arresting Hemorrhage;" A. H. Krull, "Influenza;" W. G. Huyett, "Pulmonary Emphysema;" A. P. Laubach, "Pleuro-pneumonia;" D. Allen, "Potassium Bromide."

Communications: A. D. McLauchlin, "Rumenotomy;" A. Jordan, "Fracture of Premaxilla;" E. C. Elwes, "Canker in Foot;" L. T. Dunn, "Myorrhexis;" C. W. Fisher, "Phymosis in Boar;" E. T. Cunningham, "Rupture of Stomach;" Hamlet Moore, "Azoturia;" H. W. Stedman, "Puncture of Foot;" F. M. Hayward, "Capped Elbow;" G. P. Haytes, "Prolapsus Ani;" G. W. Higginson, "Foreign Body in Posterior Femoral Region;" B. Royer, "Tympanitis in Ox;" A. I. Sorensen, "Mammitis in Sow;" W. L. Adams, "Choking;" T. Rowland, "Tympanites in Ox;" W. R. Clark, "Acute Laminitis."

C. W. FISHER,
Secretary.

